# Design and Implementation of a Pressure Monitoring System Based on IoT for Water Supply Networks

**DOI:** 10.3390/s20154247

**Published:** 2020-07-30

**Authors:** José Pérez-Padillo, Jorge García Morillo, José Ramirez-Faz, Manuel Torres Roldán, Pilar Montesinos

**Affiliations:** 1 Department of Agronomy, University of Córdoba, Campus Rabanales, Ed. Da Vinci, 14071 Córdoba, Spain; jgmorillo@uco.es (J.G.M.); pmontesinos@uco.es (P.M.); 2 Department of Electrical Engineering, University of Córdoba, Campus Rabanales, Ed. Da Vinci, 14071 Córdoba, Spain; jramirez@uco.es; 3 Department of Applied Physics, University of Córdoba, Campus Rabanales, 14071 Córdoba, Spain; fa1torom@uco.es

**Keywords:** digitalization, information and communication technologies (ICT), low power wide area network (LPWAN), Arduino microcontroller, ThingSpeak, web service

## Abstract

Increasing the efficiency of water supply networks is essential in arid and semi-arid regions to ensure the supply of drinking water to the inhabitants. The cost of renovating these systems is high. However, customized management models can facilitate the maintenance and rehabilitation of hydraulic infrastructures by optimizing the use of resources. The implementation of current Internet of Things (IoT) monitoring systems allows decisions to be based on objective data. In water supply systems, IoT helps to monitor the key elements to improve system efficiency. To implement IoT in a water distribution system requires sensors that are suitable for measuring the main hydraulic variables, a communication system that is adaptable to the water service companies and a friendly system for data analysis and visualization. A smart pressure monitoring and alert system was developed using low-cost hardware and open-source software. An Arduino family microcontroller transfers pressure gauge signals using Sigfox communication, a low-power wide-area network (LPWAN). The IoT ThingSpeak platform is used for data analysis and visualization. Additionally, the system can send alarms via SMS/email in real time using the If This, Then That (IFTTT) web service when anomalous pressure data are detected. The pressure monitoring system was successfully implemented in a real water distribution network in Spain. It was able to detect both breakdowns and leaks in real time.

## 1. Introduction

Increasing the value of water as much as possible, especially in the arid and semi-arid zones of southern Europe [1] means improving the performance of water distribution networks. These regions frequently suffer prolonged drought periods [2] and this makes it increasingly difficult to secure and maintain sufficient quantities of drinking water, sometimes limiting water use in the area. A high percentage of the water distribution networks that operate in large cities have leaks that cause significant water losses. These inefficiencies are contrary to the European Commission’s reference document [3], which declares that water is a resource that must be managed sustainably and reasonably.

The current management models of water distribution systems aim to control the main hydraulic variables and ensure that these infrastructures are used efficiently. The decision-making process is based on objective data [4]. For this reason, numerous technologies have been developed to monitor hydraulic and water quality variables. Substantial progress is now being made in the development and implementation of intelligent water meters (digital water metering) based on an electronic core [5] and different communication systems to perform remote accurate readings [6]. Several studies have focused on the benefits of these meters [7], which can be improved with a complementary set of pressure sensors located in optimal positions [8,9] to detect and locate leaks [10,11]. These sensor networks have led to the development of algorithms for real-time leak detection [12]. These advances have been implemented in pilot tests in water supply companies in order to check the reliability of these technologies and any issues related to their implementation [13,14,15].

Digitization and the implementation of the concept “Industry 4.0” has created a new dimension in the management of hydraulic networks. The control of hydraulic parameters through the installation of sensors requires the storage and analysis of a large amount of data [16,17], opening the door to big data for water supply companies [18]. These companies can obtain numerous benefits as a result of the correct analysis of the stored data, such as knowing the state of the infrastructure in real time [19], detecting uncontrolled water leaks or losses [20,21], the ability to bill their clients more accurately and periodically [22], and predict consumption to anticipate possible problems [23,24].

The success of current monitoring systems applied to water distribution networks is based on the adoption of new technologies related to the Internet of Things (IoT) [25]. The IoT allows wireless connectivity between elements of the system in order to process data and make the best decision [26]. Conventional monitoring systems based on SCADAs [27], PLCs [28], etc., have been replaced by modern IoT systems, and have moved from centralized systems to decentralized systems where data are accessible from anywhere with an Internet connection. According to [29] the IoT applied to water management helps to increase productivity without cost increases, improves system efficiency and fosters the creation of new business models based on full control of the network.

The communication networks best suited to water supply systems are low-power wide-area networks (LPWAN) [9]. The companies responsible for these supply systems generally have some commonalities: hydraulic infrastructures that occupy a large surface area, work areas with little or no mobile coverage, very diverse topology that makes it difficult to access certain points, etc. LPWANs can be easily adapted to different scales, which facilitates their implementation in water supply networks, therefore, the correct functioning of the system is not constrained by a possible future extension of the network or an increase in the monitoring variables.

LPWAN networks are characterized by low energy consumption and low cost, and they allow for connection between far-away objects. This telecommunication system fits perfectly with applications that require little transmission information [30]. This communication architecture is now implemented frequently due to the ease of its development, the online available resources, and the existence of a community of developers who share their projects and results. The low cost of developing these wireless networks has led to an increase in their use, and they are favored by the growing number of compatible modules offered by major companies in the electronics sector [31]. The LPWAN networks that are currently used are Sigfox, LoRaWAN and NB-IoT.

In recent years, the use of open source technologies in sensing projects has increased for two main reasons: low component prices and the easy use of these technologies [32]. Open source platforms allow the development of large monitoring and telemetry projects at an affordable price. Technological obsolescence can be avoided because they are versatile and able to install new improvements to update libraries or improve the codes. Additionally, there is a growing community of developers (makers) who facilitate rapid self-learning due to the large amount of online resources. There are several examples of the application of open source platforms to solve real problems including a hydrological monitoring system based on Arduino Mega [33], an intelligent irrigation system that uses an IoT sensor network with Arduino Uno [34] and the monitoring of water quality with wireless communication using Arduino Leonardo [35].

To ensure the success of a monitoring system, it is necessary to properly store the data that is generated [36]. Currently, low-cost cloud databases (IoT platforms) are emerging and these require low complexity in order to facilitate their manipulation among users [37]. In addition to their main function, the orderly storage of information, they provide other secondary services such as friendly data visualization, the generation of results reports, search for patterns that follow similar behaviors, data analytics, etc. [38]. Hence, it is necessary to use these tools to manage large amounts of data and information.

The main goal of this research was to develop a low-cost and robust system for pressure monitoring in water transmission systems to detect leaks and breaks in real time. This system requires a measuring and signal transmission device that must be able to operate in locations without connection to the grid and the number of these devices should not affect the operation of the monitoring system. For this purpose, a low power wide area network (LPWAN) was used with a communications system based on the internet of things (IoT). The system was designed to use open source software as well as low-cost hardware in all its functions (data collection, data transmission and data visualization). This feature makes it easy to adapt to any water supply systems and to replace both software and hardware elements with the most appropriate alternatives for each situation, thanks to the system’s modular architecture. In comparison with other conventional pressure monitoring systems, the proposed system allows pressure data with a high frequency (fundamental characteristic to develop an alert system in real time) to be obtained; this offers great autonomy (the data collection device manages the optimal energy needed for its operation). The reliability of the developed prototype was tested in a real water transmission network to detect and locate leaks in real time, reduce the duration of system failure periods and maintain costs (improving its efficiency), which are key factors in the optimal management of water distribution systems.

This manuscript is structured as follows. Section 1 introduces the problem of the inefficiency of water distribution networks and presents studies related to the monitoring of hydraulic variables. Section 2 describes the architecture and components of the proposed pressure monitoring system, as well as their operation. Then, the case study is presented in Section 3. It characterizes an actual water distribution network where three measuring devices have been tested. Section 4 shows the actual results of leak detection under different working conditions, followed by a discussion of the strengths of the proposed system versus other commercial systems (Section 5). Finally, the conclusions are provided in Section 6.

## 2. Material and Methods

**Figure 1:**
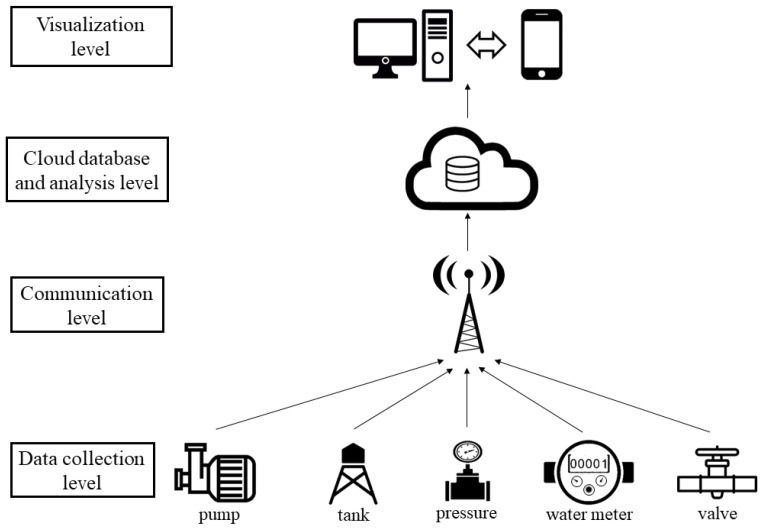
Typical low-power wide-area (LPWAN) network architecture for water supply systems.

The architecture of LPWAN networks is simple: the final nodes collect the data recorded by the sensors and connect directly to the base station to transmit the information and store it in a cloud database, where the data are analyzed and then sent to be visualized by internet-connected devices (computers, tablets or mobile phones) (Figure 1). This architecture makes the system modular. The data acquisition nodes work independently, so the installation of a larger number of nodes does not affect the behavior of the system. Another advantage is the low power consumption of each node, which improves the autonomy of the connected devices. This type of communication network allows limited transmission of information bytes, but enough to guarantee the detection of water leaks.

This type of architecture has four levels:

- Data collection level (DCL). This is the basic level of the system. This level contains the sensors that transform hydraulic variables into electrical signals. It is possible to measure the water level in the tanks, the pressure and flow inside pipes, the state of valves, the number of operating pumps in a pumping station, etc. The collection data nodes, where information is recorded and sent are independent from each other.
- Communication level (CL). The communication system chosen must be suitable for the application of the information recorded in the sensors. LPWAN networks are oriented to communicate distant elements using a narrow band. This has the advantage of using very little energy during the transmission process. Each device forms an independent node based on the IoT technology. In this way, the error of one node does not affect the correct operation of the others.
- Cloud database and analysis level (CDAL). The elements of storage and the interpretation of data coincide at this level. Cloud data storage allows remote access to the collected data.
- Visualization level (VL). The information is accessible by computers and smartphones/tablets.

**Figure 2:**
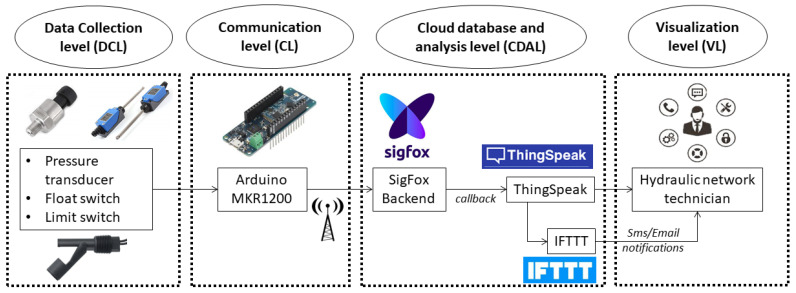
Internet of Things (IoT) pressure monitoring system (IPMS) architecture.

The following diagram (Figure 2) shows the architecture of an IoT pressure monitoring system (IPMS). The different elements that make up the system are organized in the levels described above. A detailed description of each level of IPMS is given next. The system is characterized by its low cost and the use of widely available technologies. This facilitates the replication of this system in any water supply network. The system for collecting and sending data works independently of the analysis and visualization platform, which facilitates its integration into existing monitoring systems.

### 2.1. Data Collection Level

Each node is composed of a pressure transducer that converts the hydraulic pressure into an electrical signal. Its measuring range should cover the range of possible pressures at the sensor location to ensure a reliable reading of the variable and to avoid damage to the rest of the electronics. Any change in the operational conditions of the system affects the pressure in the pipes according to the energy conservation equation between two points of the network (Equation (1)).

(1) $$\frac{P_{1}}{\gamma }+\frac{v_{1}^{2}}{2g}+z_{1}+H_{A}-H_{L}-H_{E}=\frac{P_{2}}{\gamma }+\frac{v_{2}^{2}}{2g}+z_{2}$$

where *P* is the pressure; *v* is the flow velocity; *z* is the elevation; *γ* is the specific weight of water; *H_A_* is the energy added by a pumping station; *H_L_* are friction losses in pipes and *H_E_* is the energy extracted by turbines. This demonstrates that leaks or breakdowns in the water supply network (modify v in Equation (1)) will give pressure readings out of their ordinary range at downstream points. This monitoring system will detect and locate leaks in real time, thus improving the hydraulic performance of the system. The pressure measurement is also used to understand the behavior of the hydraulic system in different operation scenarios, in order to improve the management of adverse situations.

A Honeywell transducer (PX2AN1XX250PSACX model) was used to measure the pressure. This device converts the pressure into a voltage signal ranging from 0.33 V to 2.97 V. The supply voltage is 3.3 V and the supply current is 4 mA, being fully compatible with the output voltage of most microcontroller boards that exist on the market. It also has an IP69K degree of protection, which allows it to be in contact with water without being damaged. The working range of this transducer ranges from 0 bars to 17.23 bars, which covers the normal operating pressure range in the case study network.

In addition, two sensors were included in the pressure measuring node to make it robust. As electronics will be set inside a box, these sensors are used to control its status. A limit switch will send alarms in case of theft of electronics material and a float switch will also send alarms if the box is flooded, which may occur in nodes located in the lowest elevation points of the network.

### 2.2. Communication Level

Communication Network:

The chosen solution is based on the use of Sigfox network operator. Sigfox is a LPWAN and it allows for both sending and receiving messages from any Sigfox device. In this case, one-way communication is used, i.e., only sensor readings will be transmitted. The 140 daily messages allowed by the Sigfox network are sufficient to monitor the pressure evolution in water supply networks, and data are obtained every 11 min.

The main reason for choosing this communication system is its ease of application. The Sigfox network is used as a service. The customer pays per year and per connected device. Users do not have to install and maintain a communication network. Therefore, this wireless communication system is suitable for water management companies that cover a large area, and they can avoid the major investment required to implement and maintain their own communication network.

It is important to study the coding structure of the messages transmitted by Sigfox network in order to minimize the space required [39]. This strategy reduces the time of transmission of the message, thus increasing the autonomy of the device. Long messages mean long radio signals, and this implies greater use of the battery. The size of each section is determined by the number of circumferences (4 bits).

**Figure 3:**
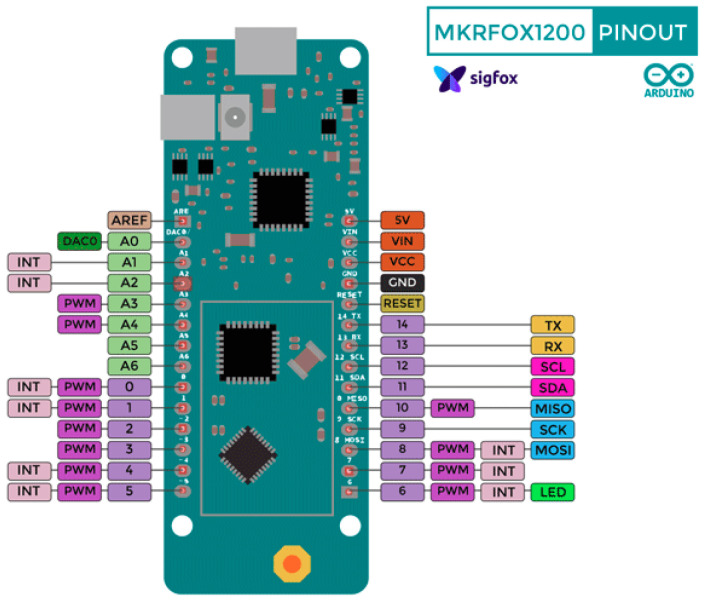
Arduino MKR FOX 1200 pinout scheme.

Hardware platform:

The hardware platform chosen to build the prototype was the Arduino MKRFOX 1200 [40]. It can be connected to the Sigfox network with a free subscription for a period of one year. This board consists of the Atmel SAMD21 microcontroller and the ATA8520 radiofrequency module with Sigfox connection. The main specifications are: 48 MHz speed, 256 Kb Flash memory and 32 Kb SRAM memory, 28 digital pins and 10 analog pins, and 3.3 V power supply. Figure 3 shows the layout of the output pins on the board chosen for this project, the Arduino MKR FOX 1200.

**Figure 4:**
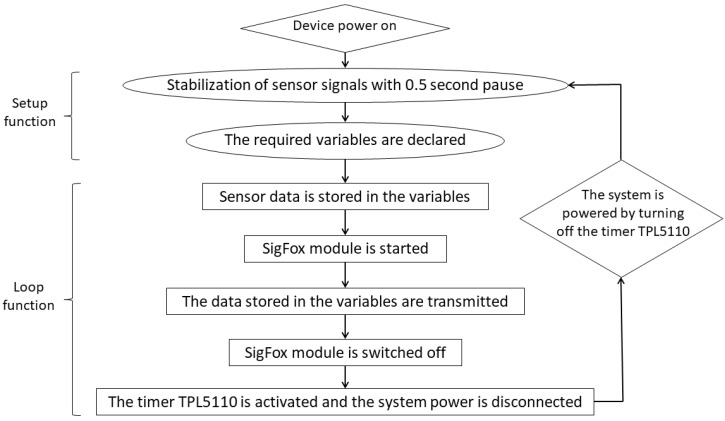
Activity diagram showing the functions performed by the Arduino microcontroller.

The Arduino microcontroller collects the information provided by the sensors and periodically sends data via the Sigfox network. To do this, it has been programmed in C++ language through its integrated development environment (IDE) following the activity diagram shown in Figure 4.

Arduino does not perform data storage functions. It just reads the information generated by the sensors and transmits it. The data are stored together in the cloud with data from other nodes. The designed code is aimed to send information efficiently with low energy consumption and minimal execution time. After two consecutive cycles, the energy supply to the microcontroller is interrupted by the timer to extend the autonomy of the batteries.

Timer:

As the Arduino MKR1200 board has a deep sleep consumption of approximately 12 mA, it is necessary to equip the prototype with a low power timer like TPL5110 to save battery power during each sending cycle. This element is placed between the energy supply source and the rest of the components. This element aims to cut the current supply to the whole device, thus avoiding energy consumption by the rest of the components between sending cycles.

The TPL5110 allows power interruptions in periods ranging from 100 ms to 7200 s, and it consumes 35 nA during these periods. The duration of each interruption is determined by the value of the resistors connected to a timer pin. The TPL5110 is able to manage a voltage range between 1.8 V and 5.5 V (which is compatible with the Arduino MKR FOX 1200 board that operates at 3.3 V).

Battery:

Li-ion 18650 rechargeable batteries were chosen as the supply source. These batteries have a nominal voltage of 3.7 V and a capacity of 2900 mAh [41]. This type of battery is ideal for IoT devices because they have a low level of self-discharge and they require minimum maintenance.

The nominal voltage of the Li-ion 18650 batteries is consistent with the operating voltage of the Arduino MKR FOX 1200 board (up to 6 V via the VIN pin), the supply voltage of the pressure transducer (3.3 V) and the operation of the TPL5110 timer (1.8 V–5.5 V). For this reason, it is not possible to install several batteries in series. A set parallel cells was used in order to increase the capacity and improve the autonomy of the device.

**Figure 5:**
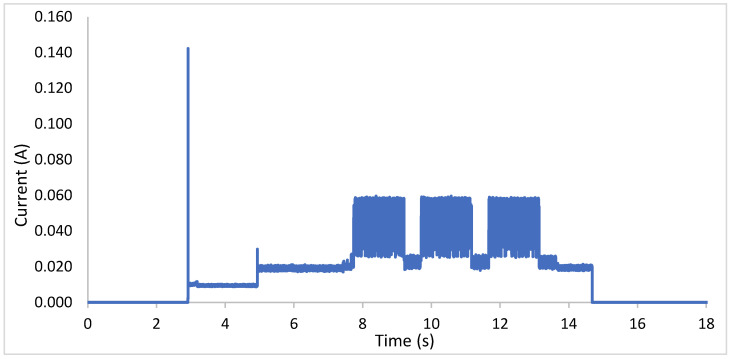
Device consumption per data transmission cycle.

Autonomy:

The device´s consumption was monitored using a YOKOGAWA DL850E oscilloscope (Figure 5). It is necessary to know its consumption during each data transmissions cycle to estimate the battery autonomy. Each transmission period has a duration of 12 s in which the device goes through four different stages:

- Timer on: this is the status of the device between message sending cycles. The intensity during this period is 0.05 mA because only the timer TPL5110 uses energy.
- Peak consumption: this occurs when the microcontroller starts the Sigfox connection module with a peak intensity of 0.14 A.
- Signal stabilization: this period always last 2 s during which the signal is stabilized for a correct reading of the sensor data. In this period, the intensity of the prototype reaches 0.010 A.
- Sending information: this period varies according to the length of the message. The duration of the sending is approximately 10 s while the current ranges between 0.020 A and 0.060 A.

In order to quantify the number of Li-ion 18650 batteries, which are arranged in parallel and required to provide the device with reasonable autonomy, the following sensitivity analysis was carried out. Table 1 shows the autonomy of the device according to the number of cells arranged in parallel, the frequency of sending messages and the electric consumption at each stage of data transmission.

The results obtained in the theoretical simulations of the autonomy of the device are gathered in Table 1 and show that the best option is to connect four batteries in parallel. The set of four parallel cells has a total capacity of 11,600 mAh and the autonomy of the device is up to 3 years, sending information every 15 min (which is suitable for monitoring pressure in water supply systems). After this period, the batteries must be replaced by fully charged batteries. Autonomy is a key issue when IoT devices are scattered over large areas, as in the case of water transmission systems. This dispersion combined with the inaccessibility of certain monitoring points requires maximizing the battery lifespan for the applicability of the device.

**Figure 6:**
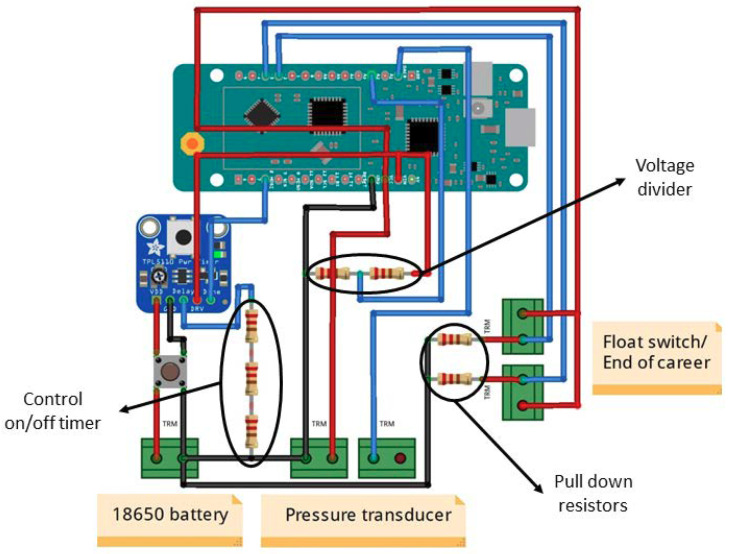
Prototype printed circuit board (PCB) wiring diagram.

Control circuit module:

Figure 6 shows the connections between the different elements that compose the printed circuit board (PCB). Five terminal blocks were arranged to facilitate the connection of the sensors and the battery. A nano-switch was also installed to disconnect the power supply for repair or maintenance of the device.

The resistors shown in Figure 6 have various functions. There are three resistors arranged in series to control the timer on/off. The two resistors connected in series form a voltage divider to measure the battery level (4.2 V at maximum capacity), without damaging the Arduino board (input of 3.3 V maximum). Finally, the two remaining resistors are pull down resistors, which establish a logic state (LOW) on an Arduino input pin when it is on standby to avoid false input pin states caused by noise generated in electronic circuits.

**Figure 7:**
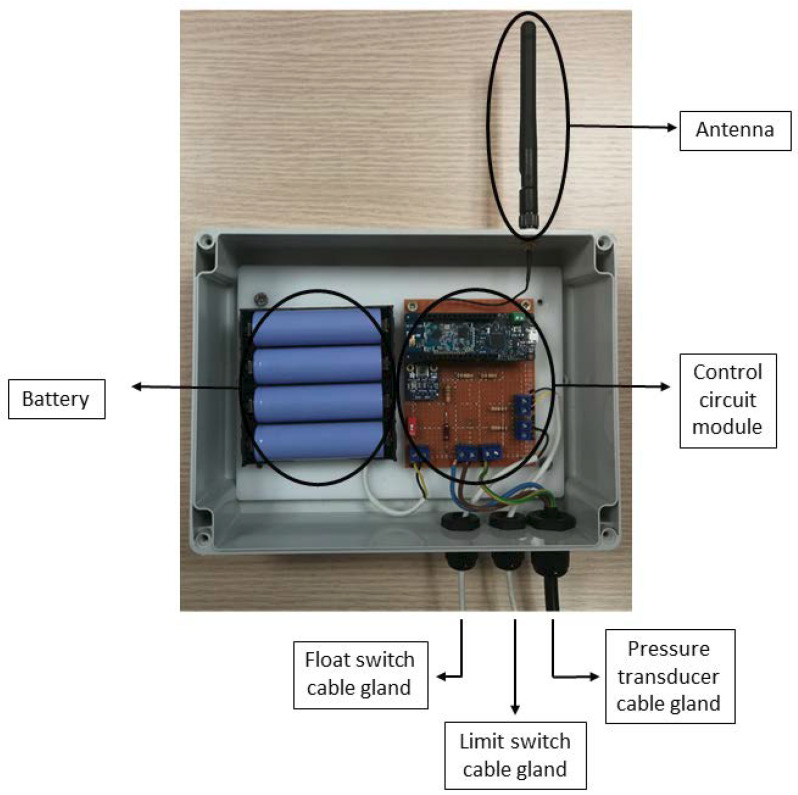
Internet of Things pressure monitoring system (IPMS) electronics.

Register box:

All the elements of the prototype were placed in a waterproof register box to protect and isolate them from the adverse weather. The box has an IP65 degree of protection to guarantee the device durability (Figure 7). Three cable hoses were used to connect the PCB and the sensors. Each cable exits the box through a cable gland to maintain the water-tightness of the prototype.

To improve the quality of the radio signal, the antenna was placed outside of the register box using a rubber gasket to maintain the water-tightness. An adapter was used to convert the ufl output from Arduino to sma to facilitate the installation of an antenna that best suits the location of the device. The antenna is the only element that affects the quality of Sigfox connectivity, hence its importance in the device. For this specific case, an omnidirectional antenna with8 dB power was used.

### 2.3. Cloud Database and Analysis Level

Sigfox backend:

As described above, the maximum amount of information that can be transmitted per message is 12 bytes, and up to 140 messages per day and per node can be sent. The information included in each message is as follows:

- Battery level: this parameter varies between 3.2 V (lower limit) and 4.2 V (upper limit). The lower limit is determined by the Arduino power supply voltage. The upper limit is determined by the maximum capacity of the battery. This value is transmitted as an unsigned integer in decivolts to increase its accuracy. This variable controls the replacement of the batteries to prevent the device from not sending information.
- Pressure: indicates the pressure of the water inside the pipe. The measurement range is between 0 bar and 17.23 bar. The data is transmitted as an unsigned integer in centibars.
- Cover status: indicates the status of the manhole cover where the pressure transducer is installed. The data is transmitted as an unsigned integer with values of 0 (open door) and 1 (closed door).
- Flooding: indicates the state of the manhole where the pressure transducer is installed. The data is transmitted as an unsigned integer acquiring values of 0 (good conditions) and 1 (flooded sump).

Table 2 summarizes the measured variables and the type of data used for transmission via the Sigfox network.

The data from the different nodes installed in the network are collected in the Sigfox backend. This platform is a tool to manage messages and check that the communication is correct but it is not able to visualize the data. The information is received in a hexadecimal format.

To facilitate the display of data, callbacks redirect the data from the Sigfox backend to an alternative storage and visualization platform. In this case the ThingSpeak platform was used. The callbacks are made by identifying the URL of the ThingSpeak platform in the Sigfox backend, pointing out the variables to be transmitted. So, every time a message arrives at the Sigfox cloud, the chosen data are redirected to the ThingSpeak platform where they can be easily managed and displayed.

ThingSpeak platform:

ThingSpeak is an IoT platform developed by MathWorks to receive, visualize and analyze data in the cloud. This platform is very intuitive, so it is widely used. It has two versions: the free version that connects up to four devices and a maximum of 8200 messages/day; and the paid version can be adapted to the needs of each client depending on the number of connected devices and the number of messages per day.

This work is based on the free version. The messages are received via REST API calls and stored in the cloud database. The data are accessible from any device with an internet connection. In addition, ThingSpeak allows the use of the following modules to perform extra functions:

- Matlab Analysis: to analyze the information received using Matlab tools.
- TimeControl: this plugin is used to program actions in a regular schedule, relaying the performance of some functions with the data on other modules. For example, every 30 min an analysis is programmed to detect pressure drops with Matlab Analysis.
- React: this module continuously analyzes the input data to perform actions when events occur. This module analyses the data to send an alarm when there is loss of data due to coverage problems
- ThingHTTP: it uses the HTTP communication protocol to transmit messages to other devices or web services.

**Figure 8:**
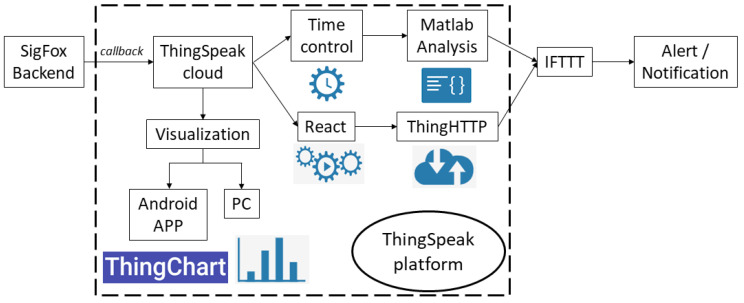
ThingSpeak platform functions in IPSM.

**Figure 9:**
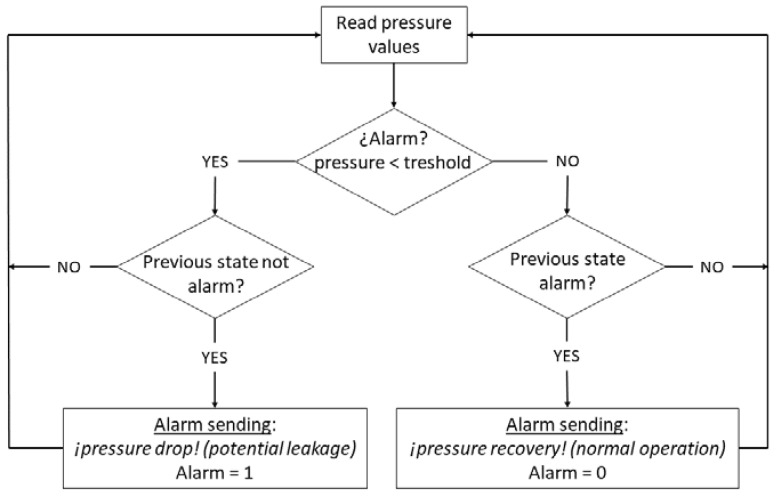
Flowchart to detect pressure variations.

Pressure information can follow two paths through the ThingSpeak platform (Figure 8):

- A quick analysis of the information can be performed with the React module based on previously defined pressure threshold values. If the pressure exceeds these thresholds, HTTP calls are made to send alerts via email to an external platform.
- For more complex analyses it is necessary to use the TimeControl module, which performs periodic analyses of historical or live data linked to the Matlab Analysis module. Matlab Analysis has great analysis potential as it uses functions from additional MATLAB toolboxes. The outcome of these analyses may prompt a HTTP call to an external platform to send warnings to the operation and maintenance personnel of water service companies. For instance, the following code is aimed at detecting sudden pressure drops and their return to normal values (Figure 9).

IFTTT (If This, Then That):

The last element in the cloud database and analysis level is the platform, ”If This, Then That” (IFTTT). This web service programs actions to automate different tasks. The user must formulate a condition and define a reaction to that condition. The objective of this tool is to identify anomalous situations from the received data and to automatically send alerts via e-mail.

The IPMS includes warnings in response to out-of-range pressure oscillations, if the manhole cover where the device is installed is opened, if the sensor box is flooded or when the battery is almost discharged. Water utility managers will receive emails with real-time information on the occurrence of these events. They receive instant information about failures that enable them to make decisions to revert these situations as quickly as possible.

### 2.4. Visualization Level

At this level, the water utility staff have access to pressure data in real time. Data must be shown in a friendly and intuitive way. The IPMS allows data consultation from any device with internet access through its website. The ThingSpeak platform also has an APP to take queries from smartphones (Figure 8). This facilitates data-based decision making in any case: for the staff in the office and for the maintenance personnel that work out of the water service offices.

The IPMS sends email warnings when anomalous system performances occur. This ensures a quick response from the water service staff. Both data and emails can be visualized on web platforms and smartphone APPs, which facilitates the communication of information to decision makers.

## 3. Case Study

**Figure 10:**
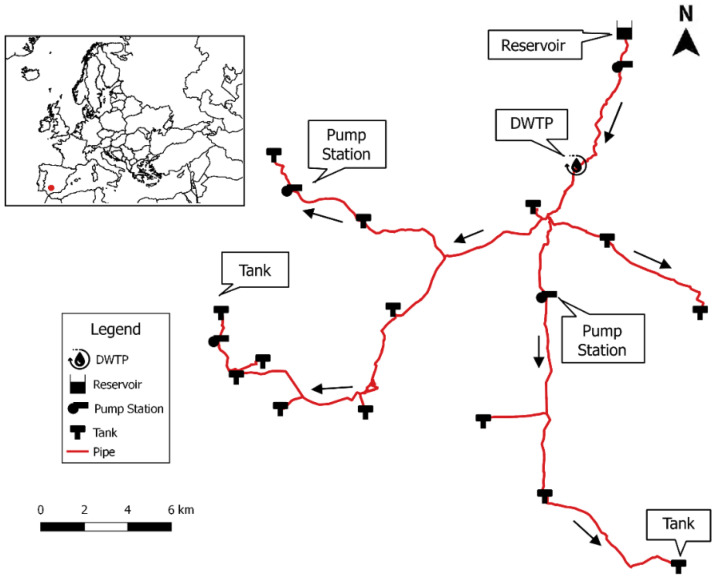
Location and layout of the hydraulic network studied.

The developed prototype was installed in the water transmission system (WTS), which supplies drinking water to the municipalities in the province of Córdoba (Spain). This WTS transports water from the Martín-Gonzalo reservoir to the municipalities located in the eastern part of the province with a population of 44,200 inhabitants in an area of about 600 km^2^ (Figure 10).

The number of inhabitants varies between 140 and 9635 in the 10 municipalities that comprise the study area. Sixty percent of the population is concentrated in three municipalities. The average water demand is 250 L/hab/day, with an average annual consumption ranging from 12,733 m^3^/year to 681,572 m^3^/year in the municipalities in the area.

The WTS consists of a total of 88.79 km of pipes of different material and diameter, as well as 18 tanks with a capacity of 80 m³ to 7500 m³, 2 pumping stations and a drinking water treatment plant (DWTP).

**Figure 11:**
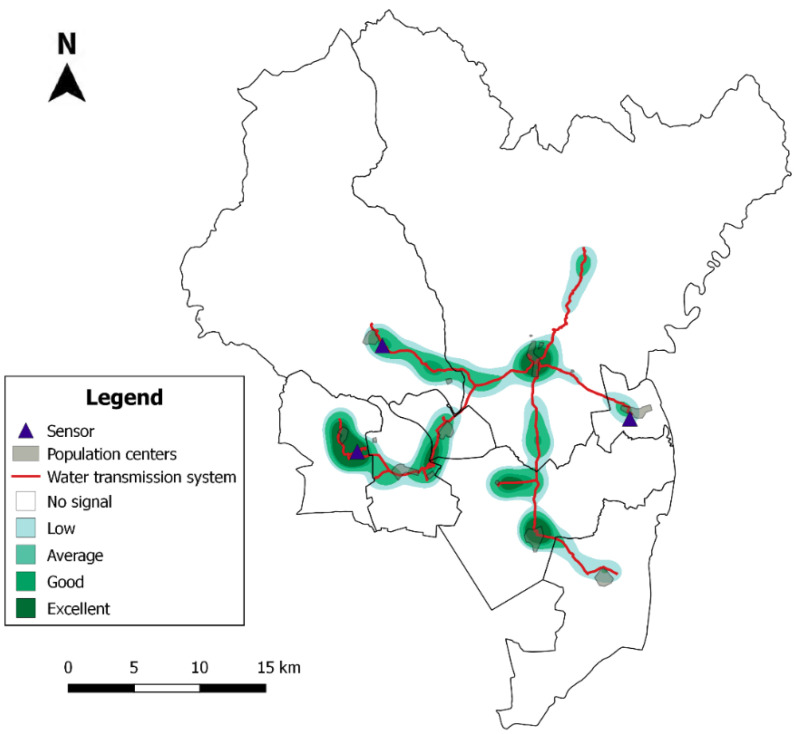
Distribution of Sigfox coverage intensity over the water distribution network.

The Sigfox signal quality in the study area was accurately measured before the sensors were installed. The results are shown in the Sigfox signal level map (Figure 11) and provide key information for choosing the optimal location for the sensors.

Figure 11 divides the signal quality into five categories: no signal, low, average, good and excellent. These categories were constructed according to two parameters: the received signal strength indicator (RSSI) and the number of stations receiving the message. The signal quality is better in areas close to towns with larger population and worse when the distance from them increases. Seventy-five percent of the WTS has acceptable coverage, allowing the Sigfox system to be used as a communication network for the designed pressure sensors.

## 4. Results

**Figure 12:**
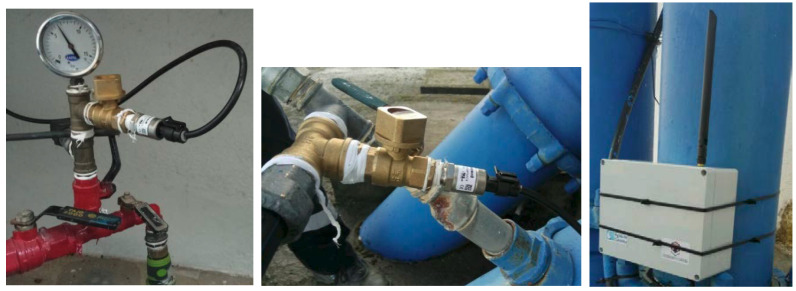
Prototype installation.

Three pressure measuring devices were installed at different locations to test the performance of the designed prototype: a tank, a pump station and an inlet (Figure 12). In each location, a valve isolates the installation point of the device from the water flow. The different locations were selected to check if the prototype works properly under different operating conditions.

Once the devices are installed, they begin to store data in the cloud database. If the pressure values are in the range previously estimated as optimal, the system will continue to store values. These can be consulted from any PC or smartphone/tablet with internet connection. In this way, it is possible to see the real-time response of the hydraulic network to the different regulation operations or the seasonal changes typical in this type of network. If there is a drop in the pressure below the defined threshold in any of the data recording nodes, the system will send an alert via SMS or email to the corresponding operators depending on the area of occurrence. Therefore, it is necessary to have the email and phone number of the responsible operators in each zone. The system tries to unify data and simplify its visualization in decentralized hydraulic networks such as transmission supply systems.

**Figure 13:**
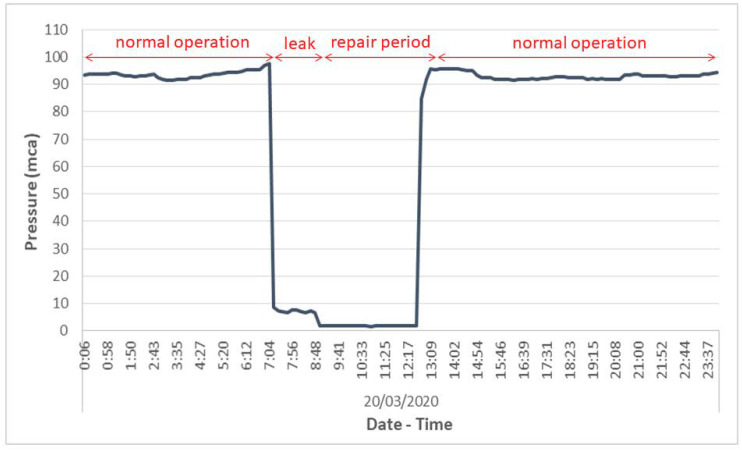
Pressure diagram in a leak event.

Leakage/breakdown detection:

Figure 13 shows the pressure data recorded at an inlet during a leakage in the main pipe of the water transmission system. A leak event consists of four stages:

- Regular operation: the recorded pressure data are in the expected range during this period.
- Leak occurrence: pressure data recorded by the sensor are not in the expected range.
- Leak repair period: the pressure recorded during the pipe fix period will be 0 due to the interruption of the water supply by the leaking pipe and the repair work is carried out.
- Back to normal operation: once the leak has been repaired, the pipe flow is restored to the usual operating pressure.

The IPMS is intended to detect the occurrence of leaks and breakdowns and locate where they occur when a warning is received from any of the sensors installed in the WTS. The rapid and accurate location of the leakage/breakdown point in pipeline networks crossing large unpopulated areas is key to leakage control. Real-time information about the pressure levels reduces the failure time (detection, diagnosis, and repair) and therefore the volume of water losses, thus improving the performance of these systems.

**Figure 14:**
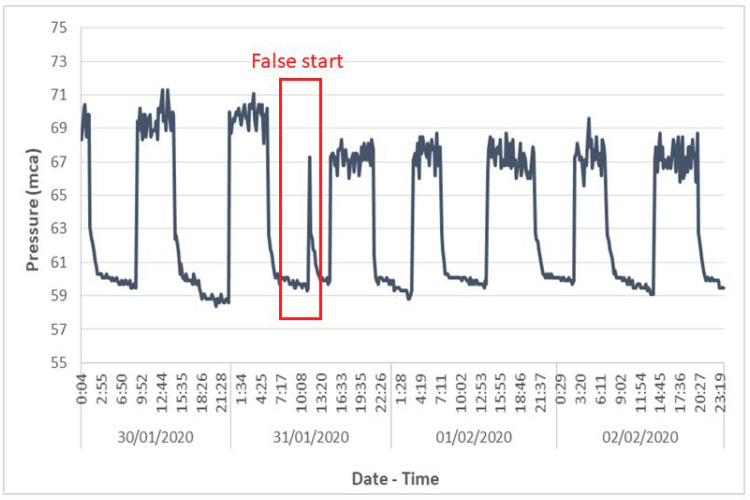
Pump false start.

Detection of anomalous pump operation:

The pumping station studied is located on a secondary pipe that supplies water to a municipal tank. The start/stop of the pump depends on the level of the tank. The pressure sensor was installed in the pipe connecting the pump to the tank. Analysis of the pressure variations allows the detection of a malfunction of the pump and the correction of possible system failures. As Figure 14 shows, false start events were detected, and were repeated at certain periods of time. This can cause long-term pump deterioration, as well as unnecessary energy consumption. By studying the pressure record, the cause of the false starts was identified, that is, an erroneous signal from the tank level sensor that sent a start command to the pump control system.

**Figure 15:**
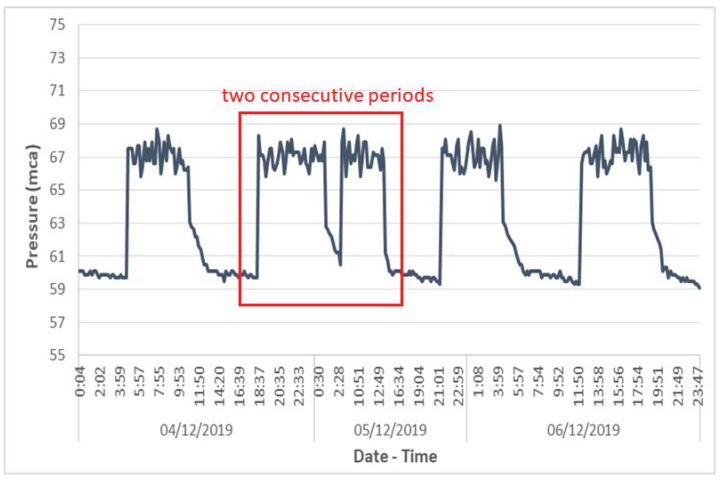
Two consecutive pump periods at a pumping station.

Analysis of the pressure evolution records in the previous pumping station also revealed other unusual operations. The pump was found to be running at one-hour intervals, when the normal duration of periods of non-operation of the pump was 7–8 h. This situation was due to the existence of a leak in the distribution system (from the municipal tank to the final consumers) with the consequent continuous drop in the level of the tank fed by the studied pumping station. Therefore, the pump had to increase its frequency of operation to compensate for leaks in the water distribution system. This anomaly was quickly detected by the IPMS (Figure 15).

IPMS costs:

The production costs of a single sensor of IPMS is detailed in Table 3. In this work, the cost of the communications system (Sigfox) was not considered as Sigfox use is free for the first year. The free version of the ThingSpeak platform was also used. Finally, the IFTTT tool is free, so there was no additional cost.

## 5. Discussion

The results confirm the adaptability of the Sigfox network for the communication of sensors located in different elements of a WTS, despite their dispersion over a large number of territories. No telecommunication infrastructure is required to use the Sigfox system, only the payment of an annual (17 € approx.) fee per connected device from the second year of use.

The proposed IoT platform, ThingSpeak has many advantages (for example, powerful advanced calculation engine, easy data visualization, online database, etc.) but the free version used in this work is limited to four devices at most. Therefore, when IPMS is composed of more than four sensors, it is necessary to have a paid subscription, whose annual cost can vary between 70 € and 580 € depending on the functions subscribed.

Compared to the existing pressure monitoring systems on the market, the proposed IPMS is 45–65% cheaper. In addition, some of the commercial pressure measurement systems often require an initial investment in telecommunication infrastructure. By using the SigFox network, this type of initial cost is avoided. According to the proposed architecture, the maintenance of servers is also avoided, which is a problem for most water service companies as they usually do not have specific staff to perform these tasks. This saves indirect costs in the implementation of the IPMS. The proposed architecture is easily scalable by increasing the number of information logging nodes. Each node acts independently, so the failure of one of them does not entail the collapse of the entire system.

The versatility of the Arduino MKRFOX 1200 microcontroller makes the proposed prototype easily upgradeable. The correction of operating errors of the device or an extension of its functions can be done by replacing the code loaded on the Arduino board. In addition, the use of this microcontroller is becoming more widespread and more detailed documentation on its operation is available. The large community of developers, along with its low cost are the main advantages in the use of this microcontroller.

Another notable aspect of the proposed prototype is its adaptability to different communication technologies. Although Sigfox technology has proven its suitability for this case, it is possible that in the near future there will be another technology more suitable for urban water supply systems. According to the layout of the control module, only the Arduino MKRFOX 1200 microcontroller should be replaced by a board that can communicate using another technology. The operation of the selected auxiliary components is not affected by the installed microcontroller.

Currently there are boards in the Arduino family that are compatible with several telecommunication technologies: WiFi (MKR 1000, MKR 1010), LoRa (MKR WAN 1300, MKR WAN 1310), GSM (MKR GSM 1400), Narrow Band IoT (MKR NB 1500) and audio and video processing (MKR Vidor 4000). All these boards have the same input and output pin layout as the chosen MKR FOX 1200 board. This would facilitate adaptation to future change in communication technology.

## 6. Conclusions

The IoT pressure sensor prototype, as an IPMS node, as well as the architecture of the communication, processing and information display system based on open source and low-cost technologies proved their applicability as a real WTS pressure monitoring and warning system. IPMS is a robust and reliable low-cost pressure monitoring system that is able to sends alerts regarding leaks, breaks and abnormal operation in pumping stations.

The system developed is versatile as the nodes can be operated in locations without connection to the conventional electrical grid with autonomy for up to 3 years thanks to the use of the TPL 5110 timer, which reduces energy consumption. The prototype, based on the Arduino MKR FOX 1200, provides data every 15 min on the pressure, the battery level and the state of the manhole where it has been installed (open/closed cover and flood warning).

IPMS architecture is modular (including information collection, data transmission, storage and display). In each module, any element and/or software can be replaced by another of similar characteristics to facilitate the updating/upgrading of the technologies used and the integration of these data into another existing telemetry system. In addition, dependence on trademarks is avoided.

The combination of low-cost and open source technologies implemented in IPMS minimizes the initial investment and maintenance costs of monitoring key hydraulic variables in the operation and maintenance of water supply services. These are very important incentives for the digitalization of the management of water supply companies, especially for companies with limited economic resources.

The evolution of IPMS in the near future could be directed to the reinforcement of its autonomy with the incorporation of a connector for photovoltaic panels in nodes in suitable locations. Also, the time between measurement cycles could be reduced by using a (non-volatile) EEPROM memory, which accumulates the data recorded in several measurements and sends them every 11 min (minimum period between sending cycles in Sigfox).

Finally, the IPMS functionality could be extended by incorporating an advanced ThingSpeak calculation module (MATLAB) to calibrate the hydraulic model of WTSs and simulate the behavior of the system under different operating scenarios based on pressure measures.

## Figures and Tables

**Table 1:** Autonomy of the device expressed in days and years depending on the number of batteries connected in parallel.

Message Frequency	Sending Consumption	Standby Consumption	1 Battery		2 Batteries		3 Batteries		4 Batteries	
(Minutes)	(mAh)	(mAh)	(Days)	(Years)	(Days)	(Years)	(Days)	(Years)	(Days)	(Years)
11	0.5304	0.05	208	0.57	416	1.14	625	1.71	833	2.28
12	0.4862	0.05	225	0.62	451	1.23	676	1.85	901	2.47
13	0.4488	0.05	242	0.66	485	1.33	727	1.99	969	2.65
14	0.4167	0.05	259	0.71	518	1.42	777	2.13	1036	2.84
15	0.3890	0.05	275	0.75	551	1.51	826	2.26	1101	3.02
20	0.2917	0.05	354	0.97	707	1.94	1061	2.91	1414	3.88
30	0.1945	0.05	494	1.35	989	2.71	1483	4.06	1977	5.42
40	0.1459	0.05	617	1.69	1234	3.38	1851	5.07	2468	6.76
50	0.1167	0.05	725	1.99	1450	3.97	2175	5.96	2900	7.94
60	0.0972	0.05	821	2.25	1641	4.50	2462	6.75	3283	8.99

**Table 2:** The variables and their format transmitted in each message.

Message	Possible Values	Category	Byte Position	Units
Battery level	0–255	uint8	0	decivolts (dV)
Pressure	0–65.535	uint16	1	centibars (cbar)
			2	
Manhole cover status	0–255	uint8	3	0 = open door; 1 = close door
Flood sensor	0–255	uint8	4	0 = good conditions; 1 = flooded manhole

**Table 3:** Production costs of the IoT pressure sensor.

Concept	Price (€)
Arduino MKRFOX1200 board	35.0
TPL5110 timer	4.5
Li-Ion Battery	12.0
Enclosure	15.0
Battery holder	2.5
Antenna	3.0
Limit switch	4.0
Float switch	2.0
Pressure transducer	83.0
Others	3.5
Total costs	164.5
